# *In vitro* caloric restriction induces protective genes and functional rejuvenation in senescent SAMP8 astrocytes

**DOI:** 10.1111/acel.12259

**Published:** 2015-02-25

**Authors:** Silvia García-Matas, Rajib K Paul, Patricia Molina-Martínez, Hector Palacios, Vincent M Gutierrez, Rubén Corpas, Mercè Pallas, Rosa Cristòfol, Rafael de Cabo, Coral Sanfeliu

**Affiliations:** 1 Aging and Neurodegeneration Unit, Biomedical Research Institute of Barcelona (IIBB), Consejo Superior de Investigaciones Científicas and IDIBAPS08036, Barcelona, Spain; 2 Experimental Gerontology Section, TGB, NIANIH251 Bayview Blvd, Baltimore, MD, 21224; 3 Department of Pharmacology and Therapeutic Chemistry, Faculty of Pharmacy, IBUB, University of Barcelona and CIBERNED08028, Barcelona, Spain

**Keywords:** astrocytes, caloric restriction, mitochondria, oxidative stress, RNA microarrays, SAMP8

## Abstract

Astrocytes are key cells in brain aging, helping neurons to undertake healthy aging or otherwise letting them enter into a spiral of neurodegeneration. We aimed to characterize astrocytes cultured from senescence-accelerated prone 8 (SAMP8) mice, a mouse model of brain pathological aging, along with the effects of caloric restriction, the most effective rejuvenating treatment known so far. Analysis of the transcriptomic profiles of SAMP8 astrocytes cultured in control conditions and treated with caloric restriction serum was performed using mRNA microarrays. A decrease in mitochondrial and ribosome mRNA, which was restored by caloric restriction, confirmed the age-related profile of SAMP8 astrocytes and the benefits of caloric restriction. An amelioration of antioxidant and neurodegeneration-related pathways confirmed the brain benefits of caloric restriction. Studies of oxidative stress and mitochondrial function demonstrated a reduction of oxidative damage and partial improvement of mitochondria after caloric restriction. In summary, caloric restriction showed a significant tendency to normalize pathologically aged astrocytes through the activation of pathways that are protective against the age-related deterioration of brain physiology.

## Introduction

Astrocytes are now known to be more relevant to brain function than once thought. Their central role in brain homeostasis (Bélanger *et al*., 2011) and their partnering with neurons in synapsis transmission (Perea *et al*., 2009) implies their involvement in all major aspects of brain health and disease. Astrocytes are intrinsically neuroprotective, but their defense mechanisms can be overwhelmed in pathological brain conditions. In normal aging, astrocytes are exposed to an increased oxidative environment that might decrease their neuroprotective capacity, as demonstrated in aged astrocyte cell models (Pertusa *et al*., 2007; García-Matas *et al*., 2008). Long-term reactive astrocytes in age-associated neurodegenerative diseases such as Alzheimer's disease (AD) lose their neuroprotective capacity and contribute to the brain pathology (Li *et al*., 2011). However, astrocytes are more resilient than neurons against age-associated pathology and a range of brain injuries. Therefore, restoring or potentiating their natural capacity for neuroprotection may be an effective strategy against neurodegeneration. For instance, the overexpression of glial cell-derived neurotrophic factor (GDNF) in hippocampal astrocytes restored cognitive loss in aged rats (Pertusa *et al*., 2008). Understanding the underlying mechanisms of response of astrocytes to antiaging therapies will help to determine the best scenario for maintaining human cognitive capacity until an old age.

Caloric restriction (CR) is the most effective antiaging therapy to date. A reduction of 10–40% in food consumption while maintaining adequate nutrients increases the average and maximum lifespan in many experimental model organisms. The exact mechanisms of CR have yet to be elucidated, but they appear to involve highly conserved mechanisms of stress response (Sinclair, 2005). CR reduces the metabolic rate and oxidative damage and induces many physiological changes opposed to the development of chronic age-associated diseases (Redman & Ravussin, 2011). However, a CR-induced increase in longevity has yet to be demonstrated in nonhuman primates (Mattison *et al*., 2012) and humans. Interestingly, CR delays the age of onset of age-associated pathologies in all species analyzed, including humans. With regard to brain function, *Rhesus* monkeys subjected to CR showed diminished age-related brain atrophy in key regions of motor and executive functions (Colman *et al*., 2009). Furthermore, a study in healthy humans showed that CR improves memory in the elderly (Witte *et al*., 2009).

Astrocytes appear to be responsive to CR intervention *in vivo*. Healthy rats showed an improvement of astroglial function in the hippocampus (Ribeiro *et al*., 2009), and transgenic mice for AD (APP + PS1) showed decreased astrocyte activation (Patel *et al*., 2005) after CR treatment. If CR is mostly a metabolic therapy, astrocytes would be the main mediators of the beneficial changes of this therapy in the brain, playing an important regulatory role in brain metabolism (Bélanger *et al*., 2011). We wished to study the changes induced by CR in senescent and healthy astrocyte cells to evaluate the usefulness of this therapy in brain rejuvenation and defense against age-associated AD-type degeneration. We chose the senescence-accelerated prone 8 (SAMP8) mouse as a source of senescent astrocytes. SAMP8 is an inbred strain obtained through phenotypic selection that presents learning and memory deficits, anxiety, impaired immune system and brain pathologies that worsen with age: mitochondrial dysfunction, oxidative stress, tau hyperphosphorylation, inflammation and amyloid-beta deposition (Tomobe & Nomura, 2009). SAMP8 mice have shown improved cognitive performance after CR treatment (Komatsu *et al*., 2008). Cultured SAMP8 astrocytes have a senescence phenotype with markers of both, aging and AD (García-Matas *et al*., 2008; Díez-Vives *et al*., 2009). Therefore, we undertook a gene expression microarray study of cerebral cortical astrocytes cultured from SAMP8 mice and its control strain senescence-resistant 1 (SAMR1) mice. To induce a CR phenotype in both, SAMP8 and SAMR1 astrocytes, we used a previously developed *in vitro* model system using serum from rodents subjected to CR (de Cabo *et al*., 2003). Treatment with CR serum will induce physiological and gene expression changes related to CR mechanisms in astrocytes cultured *in vitro*, as previously demonstrated in rodent hepatocytes and fibroblasts and diverse human cell lines (de Cabo *et al*., 2003; Hyun *et al*., 2006; Allard *et al*., 2008). We analyzed the differential transcriptomic changes induced in SAMP8 vs. SAMR1 astrocytes by CR treatment. We anticipated that many of the changes would appear in mitochondria-related pathways, as these cell organelles are hypothesized to be the main drivers of biological aging. Furthermore, mitochondria are proposed to play a significant role in pathological aging and neurodegeneration, while SAMP8 astrocytes show decreased mitochondrial membrane potential and increased generation of reactive oxygen species (ROS) (García-Matas *et al*., 2008). Therefore, we also analyzed the beneficial effects of CR against the functional alterations of mitochondria and oxidative stress of SAMP8 astrocytes.

## Results

### Caloric restriction induced a shift in the transcriptome of SAMP8 astrocytes toward SAMR1 astrocytes

The gene expression profiles of the four experimental groups of astrocyte cultures showed a significant Z score for 9709 mRNA transcripts of the 24 613 tested (39.4%). Calculation of the Z ratios for the pairs of groups showed differential expression in 1079 gene transcripts (17.5%). SAMP8 showed upregulation of 504 genes and downregulation of 312 genes in control conditions with AL treatment, compared with SAMR1 (P8AL-R1AL). CR induced major changes in both astrocyte types. CR-treated SAMR1 had 257 upregulated and 152 downregulated genes (R1CR-R1AL). CR-treated SAMP8 had 189 upregulated and 183 downregulated genes (P8CR-P8AL). Comparison of the astrocyte strains after CR treatment showed the upregulation of 446 and downregulation of 327 genes in SAMP8 compared with SAMR1 (P8CR-R1CR). See Table S1 (Supporting information) for a list of differentially expressed transcripts.

PAGE analysis demonstrated 803 pathways with altered expression in SAMP8 astrocytes compared with SAMR1 out of the 1689 tested (47.6%). CR normalized 318 of the differentially expressed pathways, namely more than a third of them. In addition, 203 new pathways showed a significant Z score after CR, but with overwhelmingly mild differences in expression. With regard to the specific effects of CR in each strain, the treatment induced more changes in SAMP8 than in SAMR1, with 807 and 664 altered pathways, respectively. These CR-induced changes on coincident pathways in the two strains were in the opposite direction of change in 53% of cases. Collectively, CR induced differential changes in the SAMP8 and SAMR1 transcriptomes and made SAMP8 astrocytes more similar to SAMR1 ones than in control conditions (Fig. S1, Supporting information). Notably, the upregulation of gene sets such as Aging brain up (Z score P8AL-R1AL: 3.9999) and AD up (Z score P8AL-R1AL: 5.0492) in SAMP8 was totally prevented by CR treatment (see Table S2, Supporting information for a complete list of pathway results).

**Fig 1:**
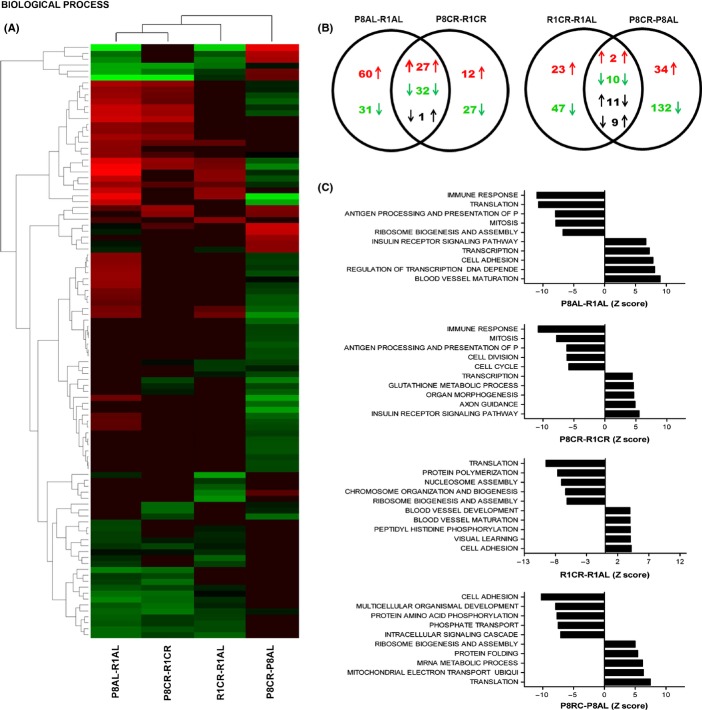
Gene pathways of biological processes were modulated differentially in SAMP8 vs. SAMR1 astrocytes, and these differences were attenuated by caloric restriction. (A) Hierarchical cluster of the 100 gene pathways of gene ontology (GO) gene sets for biological processes most differentially modulated between the experimental groups. (B) Venn diagrams showing the number of pathways upregulated (upward arrow, red number), downregulated (downward arrow, green number) or with opposed changes (black number). (C) Histograms indicating the GO terms for the top five upregulated and downregulated biological pathways between the experimental groups.

Results of the analysis of GO gene sets for biological processes are shown in Fig.1. A total of 151 GO terms differed between SAMP8 and SAMR1 (P8AL-R1AL) and 99 differed after CR treatment (P8CR-R1CR), indicating a 34% decrease. Cluster analysis for the most significant 100 GO terms showed that CR had the effect of diminishing the activity of biological processes that was more accentuated in SAMP8 astrocytes than in SAMR1 (Fig.1A). Venn diagrams demonstrated that 64% of the biological processes altered in basal conditions were not modified by CR treatment, whereas CR induced changes in opposite direction in 62% of the commonly affected biological processes (Fig.1B). Those most downregulated in SAMP8 and not restored by CR were processes associated with immunological function and mitosis (Fig.1C). Ribosomal function and protein translation were highly downregulated but protected by CR. Those most upregulated in SAMP8 compared with SAMR1 and not protected by CR were processes of transcription and insulin receptor signaling, even though differences were reduced after CR as seen by the lower Z score of the ratios (P8CR-R1CR), whereas blood vessel maturation and cell adhesion were protected. Other gene sets also downregulated in SAMP8 but restored by CR were associated with protein folding, transport of molecules (proton, iron ion, protein), response to ROS and ATP synthesis (not shown, see Table S3, Supporting information). It should be noted that glutathione metabolism was differentially upregulated in SAMP8 by CR treatment. Other gene sets upregulated in SAMP8 but restored by CR were associated with organogenesis processes, extracellular matrix organization and MAPKKK cascade signaling (Table S3, Supporting information).

The results of the analysis of GO gene sets for cellular component are shown in Fig. S2 (Supporting information). A total of 65 GO terms differed between SAMP8 and SAMR1 (P8AL-R1AL), and 35 differed after CR treatment (P8CR-R1CR), namely differences decreased by 46%. Cluster analysis of the most significant 100 GO terms showed that CR induced many changes in SAMP8 that led to small differences, mostly downregulation, of gene sets of the cellular components in comparison with SAMR1 (Fig. S2A, Supporting information). Venn diagrams showed that 54% of gene sets that differed between SAMP8 and SAMR1 in basal conditions were not modified by CR (Fig. S2B, Supporting information). Treatment also induced changes in opposite direction in 43% of commonly affected gene sets in the two strains. Many ribosome and mitochondria component gene sets were highly downregulated in SAMP8 and restored by CR (Fig. S2C, Supporting information). Some of the extracellular matrix and membrane components that were upregulated in SAMP8 were restored by CR (i.e., proteinaceous matrix, collagen, basement membrane) (see Table S4, Supporting information).

The results of the analysis of GO gene sets for molecular function are shown in Fig. S3 (Supporting information). A total of 86 GO terms differed between SAMP8 and SAMR1 (P8AL-R1AL), and 59 differed after CR treatment (P8CR-R1CR). Cluster analysis of the most significant 100 GO terms showed that CR induced many changes in the expression of SAMP8 gene sets, which decreased the differences between both types of CR-treated astrocytes (Fig. S3A, Supporting information). Venn diagrams showed that 47% of the gene sets that differed between SAMP8 and SAMR1 in basal conditions were not modified by CR. Treatment also induced changes in opposite direction in 67% of commonly affected gene sets in the two strains (Fig. S3B, Supporting information). Ribosome-related molecular functions, antioxidant activities and hydrogen ion transport were highly downregulated gene sets in SAMP8 that were restored after CR. Molecule-binding gene sets (protein, metal ion, nucleic acid) were upregulated in SAMP8 in basal conditions and were mostly restored after CR treatment (Fig. S3C, Supporting information). Extracellular matrix-upregulated gene sets were partially protected by CR; namely longitudinal stress was protected, but not structural integrity. Upregulated gene sets involved in the regulation of transcription were partially normalized by CR. Quantitative PCR data for selected genes highly upregulated or downregulated in SAMP8 are shown in Table S6 (Supporting information).

### Caloric restriction ameliorated senescence phenotype of SAMP8 astrocytes

**Fig 2:**
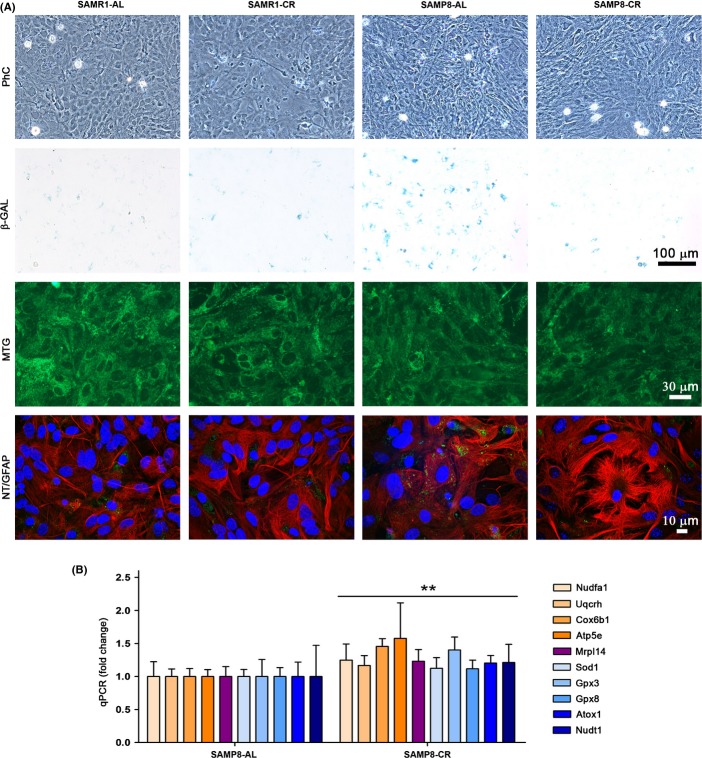
Senescence phenotype of SAMP8 astrocytes was ameliorated by caloric restriction. (A) No distinctive morphological changes were observed by phase contrast microscopy (PhC) in SAMP8 as compared to control strain SAMR1, but β-galactosidase (β-GAL) blue staining of the same microscopy fields indicated the presence of replicative senescence in SAMP8 astrocytes treated with *ad libitum* serum (SAMP8-AL), whereas staining was greatly reduced in those treated with caloric restriction serum (SAMP8-CR). Mitotracker green FM (MTG) stained a similar mitochondrial pattern in the different cultures (green fluorescence). Immunostaining of nitrotyrosinated proteins (NT) was only visible (green fluorescence spots) in SAMP8-AL astrocytes, whereas that of GFAP (red fluorescence) showed similar astrocyte morphology in the different cultures; nuclei were counterstained with TO-PRO-3 iodide (confocal images). Representative images, *n *= 4. (B) Gene expression of mitochondrial and antioxidant genes measured by real-time qPCR showed a moderate increase in SAMP8 astrocytes after CR treatment. Nudfa1, Uqcrh, Cox6b1, Apt5e, and Mrpl14 encode proteins of mitochondrial complexes I, III, IV, V, and mitochondrial ribosomes, respectively; Sod1, Gpx3, Gpx8, and Atox1 encode a variety of soluble and membrane antioxidants, and Nudt1 a selective nucleic acid antioxidant. See Table S7 (Supporting information) for full name of genes and oligomers used. Statistics: **P < 0.01 overall effect of CR treatment according to ANOVA, without *post hoc* Bonferroni's test significance.

Microscopical analysis did not show major changes of cellular morphology or mitochondrial mass in SAMP8 astrocytes as compared to SAMR1 (Fig.2A). However, SAMP8 cultures showed replicative senescence as indicated by elevated β-galactosidase activity, which was protected by CR treatment (Fig.2A). Furthermore, SAMP8 astrocytes showed accumulation of oxidative damage that is also associated to aging. Nitrotyrosinated proteins detected in SAMP8 cultures were protected by CR treatment (Fig.2A). Quantitative changes of mRNA expression were analyzed for representative mitochondrial complex and ribosome components and antioxidant molecules that were increased after CR treatment according to microarray results. These genes resulted moderately upregulated as shown in Fig.2B (ANOVA, factor treatment, *F*_1,56_ = 7.625, *P *= 0.0078; without individual significant changes) and then might help as a whole to ameliorate senescence phenotype of SAMP8 astrocytes. Gene full names and data are shown in Table S7 (Supporting information). Next, oxidative status and mitochondrial function were deeply analyzed as described below.

### Caloric restriction reduced oxidative damage of SAMP8 astrocytes

**Fig 3:**
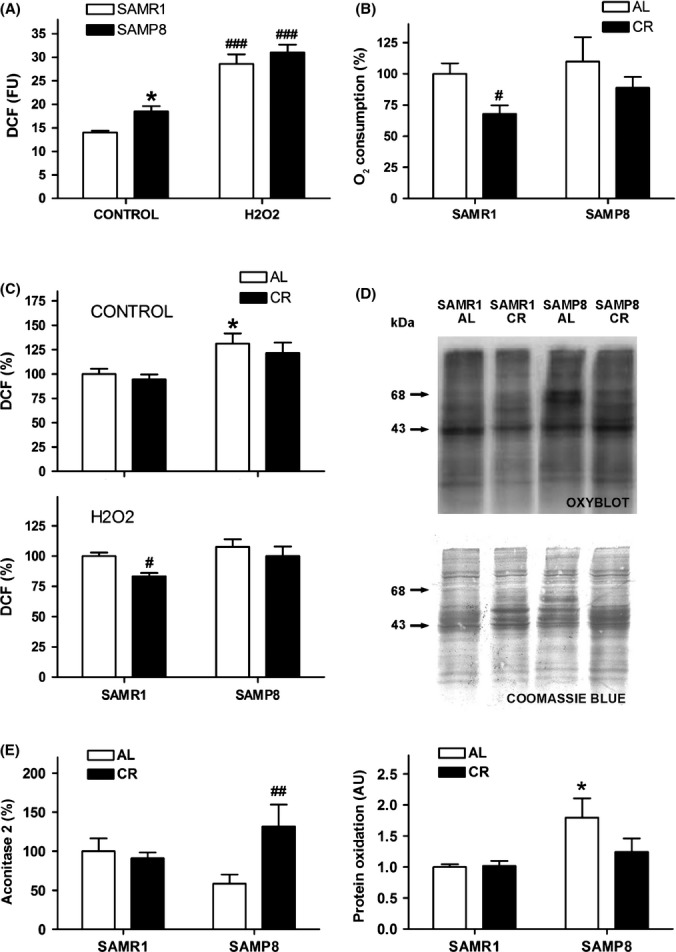
Caloric restriction reduced the oxidative stress suffered by SAMP8 astrocytes. (A) SAMP8 astrocytes generated more reactive oxygen species (ROS) than SAMR1 astrocytes in control conditions as measured by increased dichlorofluorescein (DCF) fluorescence units (FU), whereas differences between the two cell types were minor in the presence of hydrogen peroxide. (B) Caloric restriction (CR) reduced the oxygen consumption of the astrocyte cells compared with control conditions (AL). (C) CR barely decreased the excess ROS generated by SAMP8 astrocytes, whereas the decrease it induced in the ROS generated by hydrogen peroxide was only significant in control SAMR1 astrocytes. (D) CR reduced the increased level of carbonylated proteins of SAMP8 astrocytes, as shown in representative oxyblots. Coomassie blue staining was used to normalize protein oxidation in an integrated area including the two main oxidation bands (arrows). Statistics: *P < 0.05 vs. SAMR1; #*P* < 0.05, and ##*P* < 0.01, vs. control *ad libitum* (AL) conditions by Bonferroni's test.

SAMP8 astrocytes showed higher levels of ROS than SAMR1 ones (Fig.3A). Differences between the two cultures were smaller after oxidative injury caused by hydrogen peroxide, which greatly increased ROS generation as tested by DCF (ANOVA indicated an effect of strain *F*_1,63_ = 6.412, *P* = 0.0138; and hydrogen peroxide *F*_1,63_ = 98.87, *P* < 0.0001). SAMR1 and SAMP8 astrocytes showed similar levels of oxygen consumption (Fig.3B). CR treatment decreased the respiratory rate, as expected. This decrease was significant in SAMR1, whereas SAMP8 showed a clear trend with mean values similar to SAMR1 astrocytes (ANOVA indicated an effect of CR treatment, *F*_1,23_ = 6.009, *P* = 0.0222). CR reduced ROS generation in SAMR1 astrocytes (Fig.3C) (ANOVA indicated the significance of strain in control and hydrogen peroxide conditions, *F*_1,42_ = 11.24, *P* = 0.0017 and *F*_1,51_ = 6.269, *P* = 0.0155 respectively; and an effect of CR treatment against hydrogen peroxide, *F*_1,51_ = 6.072, *P* = 0.0171). The presence of oxidized proteins was significantly higher in SAMP8 astrocytes, as shown by the analysis of carbonyl groups (Fig.3D). Levels of SAMP8 oxidized proteins were close to SAMR1 values after CR treatment (ANOVA indicated significance of strain, *F*_1,32_ = 6.729, *P* = 0.0142). Levels of the mitochondrial antioxidant enzyme aconitase 2 showed a trend to decrease in SAMP8 astrocytes and a significant increase after CR treatment (Fig.3E) (ANOVA indicated an interaction of strain x treatment, *F*_1,22_ = 5.975, *P* = 0.230).

### Caloric restriction improved mitochondrial complex proteins of SAMP8 astrocytes

**Fig 4:**
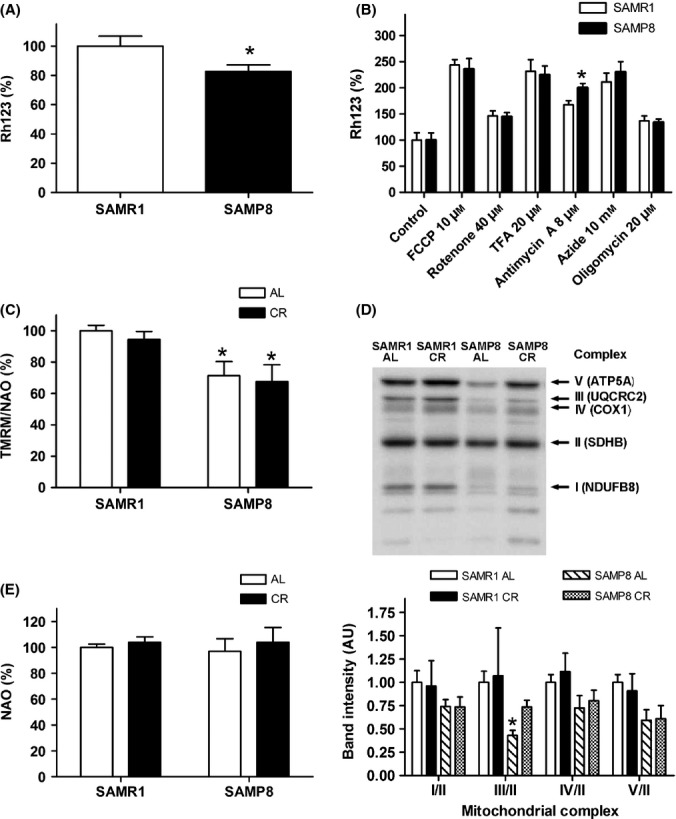
SAMP8 astrocytes showed mitochondrial impairment that was partially ameliorated by caloric restriction. (A) SAMP8 astrocyte mitochondria had reduced membrane potential (MMP) as measured by rhodamine 123 (Rh123). (B) Mitochondrial uncouplers and specific inhibitors showed the differential sensitivity of SAMP8 astrocytes against antimycin A, an inhibitor of complex III, compared with SAMR1 astrocytes. (C) Caloric restriction (CR) barely modified MMP in comparison with the control *ad libitum* (AL) treatment, measured according to the ratio of tetramethylrhodamine methyl ester to nonyl acridine orange (TMRM/NAO). (D) Mitochondrial complex proteins showed a general trend to decrease in SAMP8 in comparison with the SAMR1 levels. CR improved the level of the mitochondrial complex III protein marker in SAMP8, which was significantly decreased, as shown in representative immunoblots and densitometry analysis of proteins from the respiratory chain complexes. The immunoreactivity of the protein bands of adenosine triphosphate (ATP) synthase subunit α (ATP5A, complex V), ubiquinol-cytochrome-c reductase complex core protein 2 (UQCRC2, complex III), cytochrome c oxidase I (COX1, complex IV), and NADH dehydrogenase [ubiquinone] 1 β subcomplex subunit 8 (NDUFB8, complex I) was normalized to that of succinate dehydrogenase [ubiquinone] iron-sulfur subunit (SDHB, 30 kDa subunit, complex II) protein. (E) CR barely modified the mitochondrial mass as measured by NAO. Statistics: *P < 0.05 vs. SAMR1 by Bonferroni's test.

SAMP8 astrocytes had a lower MMP than SAMR1 astrocytes, as tested by rhodamine 123 (Fig.4A). In addition, SAMP8 astrocytes were more sensitive to the respiratory complex III selective inhibitor antimycin A (Fig.4B). Therefore, they showed a functional deficit in the respiratory complex III. SAMP8 astrocytes tested using TMRM/NAO also showed a decreased MMP in comparison with SAMR1 (Fig.4C). This difference was not ameliorated by CR (ANOVA indicated an effect of strain *F*_1,18_ = 15.20, *P* = 0.0011; and no significant effect of CR treatment). CR did not protect SAMP8 from antimycin A sensitivity either (not shown). SAMP8 showed decreased levels of markers of mitochondrial complexes as compared to SAMR1 that were significant for complex III, IV and V (ANOVA, factor strain, *F*_1,28_ = 7.295, *P* = 0.0120, *F*_1,28_ = 5.317, *P* = 0.0290 and *F*_1,28_ = 7.301, *P* = 0.0116, respectively). CR induced a trend to recover complex protein levels, mainly the most depressed complex III (Fig.4D). NAO fluorescence as indicative of mitochondrial mass did not show significant changes with CR (Fig.4E).

## Discussion

Astrocytes of the senescence mouse SAMP8 showed a gene expression profile indicative of age-related cell frailty and physiological decline. A number of genomic and proteomic studies of SAMP8 brain tissue compared with control mouse SAMR1 have been undertaken to discern the genetic basis of the complex phenotype of brain senescence that paves the path to AD or other neurodegenerative diseases. A review of the main findings concluded that SAMP8 brain shows gene expression changes in the functional categories of neuroprotection, signal transduction, protein folding and degradation, antioxidant system, energy metabolism, immune response, cytoskeleton and transport (Butterfield & Poon, 2005). The changes in gene expression in SAMP8 astrocytes found in the present study generally fit into these categories. Note that the increased expression of genes linked to pathways of both aging and AD confirms the trend for pathological aging of the SAMP8 astrocytes.

SAMP8 astrocytes were defective in the expression of immune and antioxidant response genes, in agreement with a frail aging phenotype characterized by a reduced capacity to cope with cell stressors. Frailty is induced in the process of aging by a critical lowering of the physiological reserve capacity following a lifetime of injuries. Once the condition of frailty is reached, cells are prone to age-related physiological decline (Kirkland & Peterson, 2009). For instance, accumulation of oxidative damage may lead to the exhaustion of antioxidant capacity and open the way to age-related pathologies (Salmon *et al*., 2010). Similarly, immunological response to antigen presentation is diminished in frail elderly (de la Rosa *et al*., 2006). In the brain, astrocytes have antioxidant and antipathogen activity. These two defense functions, the latter together with microglia, greatly contribute to the neuroprotective role of astrocytes. Their decline in the SAMP8 astrocytes will reduce their vital support to neurons, consistent with the results reported in a co-culture system (García-Matas *et al*., 2008).

Treatment with CR serum induced huge changes in the gene expression of astrocytes. In control astrocytes, as expected, CR reduced cell growth-related mechanisms and prepared the cells for survival in a low-nutrient environment. Similarly, a previous study with a hepatoma cell line treated with CR serum showed a gene modulation, leading to the reduction of cell proliferation and enhanced tolerance to stress (de Cabo *et al*., 2003). Notably, the effects of CR were largely opposed in SAMP8 astrocytes compared with SAMR1, leading to a reduction in transcriptomic differences between the two strains. For instance, downregulation of pathways related to protein synthesis and upregulation of genes related to extracellular matrix and to maturation of some organs such as blood vessels were observed in SAMR1. Cell growth pathways were downregulated and extracellular matrix and organ maturation were already upregulated in SAMP8 in basal conditions. CR generally ameliorated these changes toward healthy astrocyte levels, although did not reverse some of the senescence markers such as the immune deficiency shown by SAMP8 cells. Interestingly, CR reversed the substantial decrease in gene expression found for components and functional pathways of ribosomes. Indeed, a decrease in ribosome gene expression has been implicated in age- and AD-related changes (Payão *et al*., 1998), and a decreased rate of protein synthesis has been reported in aged mitochondria (Hudson *et al*., 1998). However, CR induced a downregulation of ribosome-related genes in control SAMR1 astrocytes in agreement with a reduction in protein synthesis to lower energy expenditure. Therefore, CR induced changes in opposite direction in SAMP8 cells to bring their pattern of gene activation closer to that of healthier cell conditions.

SAMP8 astrocytes showed increased ROS generation and protein oxidation, signs of accelerated aging. Accumulation of oxidative damage with age might derive from increased mitochondrial production of ROS unbalanced with the cellular response to oxidative stress (Balaban *et al*., 2005). Remarkably, CR specifically increased antioxidant defense gene pathways in SAMP8 astrocytes. Therefore, we found a decrease of protein carbonylation and nitrosylation in CR-treated SAMP8 astrocytes although their generation of ROS was not significantly decreased. Interestingly, levels of the mitochondrial antioxidant enzyme aconitase 2 were higher in SAMP8 astrocytes after CR treatment. This and other antioxidant agents would contribute to improve the redox state and decrease the oxidative damage markers. A previous report has shown a reduction of protein carbonyl and nitrotyrosine levels and of the lipid peroxidation marker 8-isoprostane in a similar *in vitro* CR model with human neuroblastoma SH-SY5Y cells (Hyun *et al*., 2006). This study had also demonstrated an increase of antioxidant defenses and a decrease of oxidative damage in the plasma membrane of rat brain submitted to CR. Other authors have reported that CR treatment *in vivo* increases the activity of the glutathione system and reduces ROS generation in rat brain (Ribeiro *et al*., 2012).

Mitochondrial dysfunctions are believed a main contributor to age-related oxidative stress and cell frailty. In this regard, the general deficiencies found in mitochondrial complexes, more noticeable in complex III, may be the basis of the senescent phenotype of SAMP8. A decrease in complex III activity has also been reported in mitochondria from whole-brain homogenates of SAMP8 in comparison with SAMR1 at 4 weeks of age (Fujibayashi *et al*., 1998). A reduced ATP content in hippocampal tissue of aged SAMP8 (Xu *et al*., 2007) confirms the progressive failure of mitochondrial function *in vivo*, although we did not found a decrease of oxygen consumption *in vitro*. The bioenergetic efficiency of mitochondria increases under CR, which leads to the generation of less ROS for equivalent ATP production than in control conditions (López-Lluch *et al*., 2006). The CR treatment *in vitro* improved the mitochondrial machinery of SAMP8, but this was not enough to normalize mitochondrial alterations such as reduced MMP. Indeed, CR has been reported to decrease MMP as part of the adaptive shift in mitochondrial energy metabolism and resistance to oxidative stress (López-Lluch *et al*., 2006). In line with higher mitochondrial efficiency, a decrease in oxygen consumption after CR treatment has been shown in rat hepatocyte and human HeLa cells (López-Lluch *et al*., 2006) and whole-body healthy (Civitarese *et al*., 2007) and obese humans (Redman & Ravussin, 2011). Metabolic adaptation in humans accounts for a 6% reduction in sedentary energy expenditure (Redman & Ravussin, 2011). There was around 30% decrease in oxygen consumption in SAMR1 astrocytes similar to a 40% decrease previously found in rat and human cell cultures (López-Lluch *et al*., 2006). A decrease also shown by SAMP8, although not statistically significant, was consistent with some adaptive response to CR-induced changes. Furthermore, CR induced rejuvenation effects in SAMP8 mitochondria, namely the partial restoration of damaged organelles through the upregulation of genes for mitochondrial components, amelioration of functional pathways and protection against further damage through the upregulation of antioxidant pathways.

The adjustment of energy expenditure and general amelioration of cellular stress responses induced by CR appears to be the basis of the cognitive improvement reported in several mouse models of aging and AD. Molecular mediators include neurotrophic factors, neurotransmitter receptors, protein chaperones and mitochondrial biosynthesis regulators (Fontán-Lozano *et al*., 2008). Astrocytes are deeply involved in brain functioning either by supporting neuron survival and plasticity or by directly participating in neurotransmission pathways. Therefore, SAMP8 astrocyte amelioration by CR treatment would suggest cognitive amelioration in SAMP8 after CR interventions *in vivo*. Indeed, improvement in the passive avoidance test outcome by SAMP8 has been described after a CR treatment (Komatsu *et al*., 2008). It has been hypothesized that CR delays the triggering of age-related disease including AD. SAMP8 mice show brain degeneration traits of AD (Pallàs *et al*., 2008) as do SAMP8 astrocyte cultures (García-Matas *et al*., 2008; Díez-Vives *et al*., 2009). Accordingly, we found that the activation of gene sets related to brain aging and to AD in SAMP8 astrocytes reverted to control values under CR treatment. In the fight against AD, CR may have additional effects by decreasing amyloid and tau pathologies (Halagappa *et al*., 2007). Some of these beneficial effects of CR are at least partially mediated through activation of the sirtuin family gene SIRT1 in neurons (Qin *et al*., 2006). However, we did not detect SIRT1 gene upregulation in CR-treated astrocytes. Similarly, other authors have neither found increased levels of SIRT1 protein in the brain of old mice submitted to CR (Barger *et al*., 2008). Accordingly, although SIRT1 has emerged as central mediator of the antiaging CR benefits (Baur *et al*., 2012), a recent report in mouse liver has demonstrated a requirement for SIRT1 but not necessarily for an increased expression of SIRT1 (Mercken *et al*., 2014).

Reduction of the senescence marker β-galactosidase after CR also indicated a rejuvenation effect in the phenotype of SAMP8 astrocytes. Detection of lysosomal β-galactosidase is associated with replicative senescence (Lee *et al*., 2006). We did not detect further senescent traits, such as enlarged and flat cell morphology or lipofuscin depots, in addition to the above discussed oxidative damage.

Previous studies carried out with CR serum treatment gave some clue on the metabolites involved in its protective effects. Namely, low levels of insulin and insulin growth factor (IGF)-1 in CR serum mediate a partial protection against hydrogen peroxide in rat hepatoma cells (de Cabo *et al*., 2003). Furthermore, IGF-1/insulin signaling is inhibited by CR in human skeletal muscle, leading to the activation of longevity and protection mechanisms (Mercken *et al*., 2013). Metabolomic approaches have shown that serum of animals submitted to CR has a distinctive metabolic profile added to a decrease of age-related changes (De Guzman *et al*., 2013). Lipid, fatty acid, and bile acid metabolism pathways were identified as responsive to CR in mouse serum by these authors. Furthermore, they found a protection of age-related loss of phospholipids involved in maintaining a low peroxidizability index of cell membranes. Therefore, CR serum contains trophic factors and metabolites that induce protective pathways through changes in the activation of many genes. The CR protective effects in SAMP8 are probably mediated through the activation of those gene pathways discussed above of ribosome/protein synthesis, mitochondria/bioenergetics efficiency, and antioxidant defense. Overall, CR serum helped senescent SAMP8 astrocytes to cope with adverse conditions and also had a rejuvenating effect.

## Experimental procedures

### Astrocyte cultures and treatment

Primary cultures enriched in astrocytes were obtained from cerebral cortical tissue from 2-day-old SAMP8 and SAMR1 mice. Mice were bred in the University of Barcelona Animal House (UB, Barcelona, Spain). The first breeding pairs were obtained from the Council for SAM Research, Kyoto, Japan, through Harlan (Barcelona, Spain). All experimental procedures were approved by the Ethics Committee for Animal Experimentation (CEEA) of the University of Barcelona, Spain. Astrocyte cultures were established as previously described (García-Matas *et al*., 2008). Experiments were routinely carried out at 21 days *in vitro*. Established astrocyte cultures of both SAMR1 and SAMP8 consisted of 85–90% astrocytes, 10–15% microglia and 0.1–1% oligodendroglia.

Sera from rats subjected to *ad libitum* (AL) diet and to CR were obtained as described for the establishment of the CR *in vitro* model (de Cabo *et al*., 2003). Serum was heat inactivated at 56°C prior to use in astrocyte culture experiments. Treatment *in vitro* was performed by adding 10% volume CR or AL serum onto the astrocyte culture medium for 48 h. The different analyses were then performed in the living cells or the cultures were collected for Western blot or mRNA microarray studies.

### Analysis of microarray data

RNA was extracted from astrocyte cultures using TRIzol reagent following the manufacturer's instructions (Invitrogen, Carlsbad, CA, USA). Samples were obtained from SAMP8 and SAMR1 astrocytes treated with AL or CR serum for 48 h (*n *= 3 independent experiments). Purified RNA was hybridized to a whole-genome expression microarray (MouseRef-8 v1.1 Expression BeadChip with 24 613 gene transcripts; Illumina, San Diego, CA, USA), following protocols listed on the Gene Expression and Genomics Unit website at the National Institute of Aging (http://www.grc.nia.nih.gov/branches/rrb/dna/index/protocols.htm). Raw data were subjected to Z normalization and tested for significant changes as previously described (Minor *et al*., 2011). For parametric analysis of gene set enrichment (PAGE), a complete set of pathways in the cell and gene ontology (GO) gene sets were obtained from http://www.broadinstitute.org/gsea/msigdb/index.jsp.

Principal component analysis was performed on the replicate average for the four groups: SAMR1-AL, SAMP8-AL, SAMR1-CR, and SAMP8-CR. These tools are part of DIANE 1.0, a program developed by V.V.P and available at http://www.grc.nia.nih.gov/branches/rrb/dna/diane_software.pdf. Details of the method are described elsewhere (Kim & Volsky, 2005).

Strain and treatment differences were evaluated through a Z ratio calculation as previously described (Cheadle *et al*., 2003). In brief, raw hybridization intensity data were log-transformed and normalized to yield Z scores, which in turn were used to calculate a Z ratio value for each gene in an experimental astrocyte culture with respect to its control astrocyte culture. The Z ratio was calculated as the difference between the observed gene Z scores for the experimental and the control groups, and dividing by the standard deviation associated with the distribution of these differences.

We analyzed the changes in gene expression in SAMP8 vs. SAMR1 (P8AL-R1AL), the effects induced by CR treatment in both strains (R1CR-R1AL and P8CR-P8AL) and the changes in gene expression in SAMP8 vs. SAMR1 after CR treatment (P8CR-R1CR). Z ratio values ≥+1.5 or ≤−1.5 were chosen as cutoff values, defining increased and decreased expression, respectively. Comparison of the gene expression between all pairs of groups selected yielded a significance of *P* < 0.05. A complete screen of gene sets belonging to 1688 pathways in the cell, and involving 6281 genes, was analyzed to check for SAMP8 cell status and CR-induced changes. In addition, changes in GO gene sets associated with specific biological processes, cellular components, and molecular functions were analyzed in a further search for age-related markers and their response to CR. A Z score value for each gene set was calculated according to the Z ratios of the corresponding genes. Again the cutoff value was 1.5 and *P* < 0.05.

### Quantitative real-time PCR

For verification of specific microarray results, we performed reverse transcription (RT) using a SuperScript III First-Strand Synthesis System (Invitrogen) with random hexamers. Then, real-time quantitative (q) PCR analysis was performed with a RT2 SYBR Green/ROX PCR Master Mix (SuperArray Bioscience Corporation, Frederick, MD, USA) using gene-specific primer pairs. GAPDH was used as housekeeping gene. Primer sequences were designed according to NCBI Nucleotide BLAST database (See Tables S6 and S7, Supporting information).

### Analysis of senescent phenotype

Morphological changes were analyzed by phase contrast microscopy of astrocyte cultures fixed with 4% paraformaldehyde (Sigma, St Louis, MO, USA) and by confocal imaging after further immunostaining with antiglial fibrillary acidic protein (GFAP, 1:500; Dako, Glostrup, Denmark), followed by Alexa Fluor 546 (1:1000; Molecular Probes, Life Technologies, Alcobendas, Madrid, Spain). Oxidative damage was detected by double immunostaining with antinitrotyrosine (1:50; Abcam, Cambridge, UK), followed by Alexa Fluor 488 (1:1000; Molecular Probes). Cell nuclei were counterstained with Hoechst bisbenzimide (Sigma). Mitochondrial mass was visualized by staining fresh cultures with MitoTracker Green FM (50 nm, 30 min; Molecular Probes). Presence of replicative senescence was analyzed with the Senescence β-Galactosidase Staining kit (Cell Signaling Technology, Danvers, MA, USA), following manufacturer's instructions.

### Oxygen consumption rate

The oxygen consumption rate of SAMP8 and SAMR1 astrocytes was determined under basal conditions, namely in the absence of inhibitors or uncouplers. The resting respiration rate was determined in cultures previously treated with AL or CR serum for 48 h. Cells were trypsinized and resuspended in DMEM. Oxygen consumption of the intact cells was monitored using a S1 Clark-type electrode of high sensitivity (Oxytherm, Hansatech, Norfolk, UK) and calculated using specific software (Oxygraph Plus software; Hansatech). Results were obtained as nmol oxygen consumed/min/10^6^ cells.

### Hydroperoxide generation

Oxidative stress in astrocyte cultures was studied using 2′,7′-dichlorofluorescin diacetate (DCFH-DA, Molecular Probes) to determine intracellular hydroperoxide generation, as the main indicator of reactive oxygen species (ROS). The analysis was performed in astrocytes seeded in 96-well dishes, as previously described in detail (García-Matas *et al*., 2010). Fluorescence induced by intracellular oxidation of the DCFH probe to 2′,7′-dichlorofluorescein (DCF) was measured after 3 h of incubation in HEPES-buffered saline. Fluorescence was normalized by the protein content of each well and expressed as a percentage of the control. All astrocytes were pretreated with AL or CR serum for 48 h. For the last 24 h, the cultures were challenged with 500 μm of hydrogen peroxide added to the media or incubated in control conditions.

### Aconitase 2 levels

Changes of the antioxidant mitochondrial enzyme aconitase 2 were determined with the Aconitase 2 Profiling ELISA kit (Abcam), following the manufacturer's instructions. Analysis was performed in mitochondrial extracts obtained with the Mitochondrial Isolation Kit for Cultured Cells (Pierce, Rockford, IL, USA).

### Mitochondrial membrane potential

A decrease in the mitochondrial membrane potential (MMP) of SAMP8 astrocyte cultures in comparison with SAMR1 ones was shown using the fluorescent probe rhodamine 123, as previously described (García-Matas *et al*., 2008). Rhodamine 123 accumulation in the mitochondria is driven by the MMP. Next, we treated the astrocyte cultures with MMP uncouplers and mitochondrial complex inhibitors for 1 h after rhodamine 123 loading. Increased fluorescence in SAMP8 compared with SAMR1 cultures indicated a higher reduction of MMP induced by a higher sensitivity to the mitochondrial toxin. Results were calculated as fluorescence units per μg of protein per well and are shown as percentage of the control.

In another set of experiments, we aimed to confirm SAMP8 mitochondrial impairment using the alternative probe tetramethylrhodamine methyl ester (TMRM, Molecular Probes) and to study the effects of CR treatment. TMRM accumulation in mitochondria is also dependent on the MMP and may have some advantages over rhodamine 123 for rapid *in situ* quantitative determination. The reduced hydrophobic character of the TMRM molecule improves its dynamics. We simultaneously determined the mitochondrial mass content using nonyl acridine orange (NAO, Molecular Probes). NAO binds to cardiolipin in the mitochondria. Cultures in 96-well plates were previously treated with AL or CR serum for 48 h. Then, cells were washed with HBSS and loaded with 1 μm of NAO. After 5 min of incubation at 37°C, cultures were loaded with 10 μm of TMRM and incubated for another 20 min. Finally, cultures were washed, and the fluorescence of both probes was measured at 535 nm excitation/590 nm emission and 490 nm excitation/535 nm emission, for TMRM and NAO, respectively.

### Western blotting

Cultured astrocytes were washed in cold PBS and lysed in ice-cold RIPA buffer (10 mm PBS, 1% Igepal AC-630, 0.5% sodium deoxycholate, 2% sodium dodecyl sulfate) containing a protease inhibitor cocktail (Complete tablets; Boehringer Mannheim, Mannheim, Germany), 1 mm sodium orthovanadate, and 5 mm sodium fluoride. The homogenates were centrifuged at 4°C, and the supernatants used to evaluate protein expression by Western immunoblotting as described (García-Matas *et al*., 2008). Protein extracts were probed with a cocktail of five antibodies against selected proteins of mitochondrial complexes (MitoProfile Total OXPHOS Rodent; Abcam). Protein extracts were also tested for oxidized proteins using an OxyBlot Protein Oxidation kit (Chemicon International, Temecula, CA, USA) following the manufacturer's instructions. Protein loading was normalized by staining membranes with Coomassie blue. Densitometrical analyses were performed with Quantity One software (Bio-Rad, Hercules, CA, USA).

### Statistics

For the microarray analysis, the significance of Z score values was tested using jmp 6.0 software, included in the diane 1.0 program (see microarray methods). All the other results were expressed as mean ± SEM. Where CR vs. AL treatment was assayed in SAMR1 and SAMP8 cultures, data were analyzed by two-way ANOVA followed by *post hoc* Bonferroni's test. Paired data were analyzed with Student's *t*-test and correlations with Pearson test. These statistics were performed using graphpad prism software (v4.02; La Jolla, San Diego, CA, USA).

## Supporting Information

**Fig. S1** Histograms of Parametric analysis of gene-set enrichment (PAGE).

**Fig. S2** Gene pathways of cellular components.

**Fig. S3** Gene pathways of molecular functions.

**Fig. S4** Correlation analysis between Z ratio and qPCR fold-changes of selected genes.

**Table S1** Raw data of gene results: Summary of genes level 2 by z test of 1 signal ANOVA.

**Table S2** Raw data of Parametric Analysis of Gene-set Enrichment (PAGE) results: PathwayScoreList.

**Table S3** Raw data of Gene Ontology Term: biological_process_GOTerm.

**Table S4** Raw data of Gene Ontology Term: cellular_component_GOTerm.

**Table S5** Raw data of Gene Ontology Term: molecular_function_GOTerm.

**Table S6** Real-time qPCR validation of microarray selected data.

**Table S7** Real-time qPCR validation of mitochondria and antioxidant genes.
