# Supplementary material for: In vitro caloric restriction induces protective genes and functional rejuvenation in senescent SAMP8 astrocytes
Source: Aging Cell. 2015 Feb 25;14(3):334–44. doi: 10.1111/acel.12259 (PMC4406662; doi:10.1111/acel.12259)
Supplement: Supplementary file 1 [file acel0014-0334-sd1.docx]

**
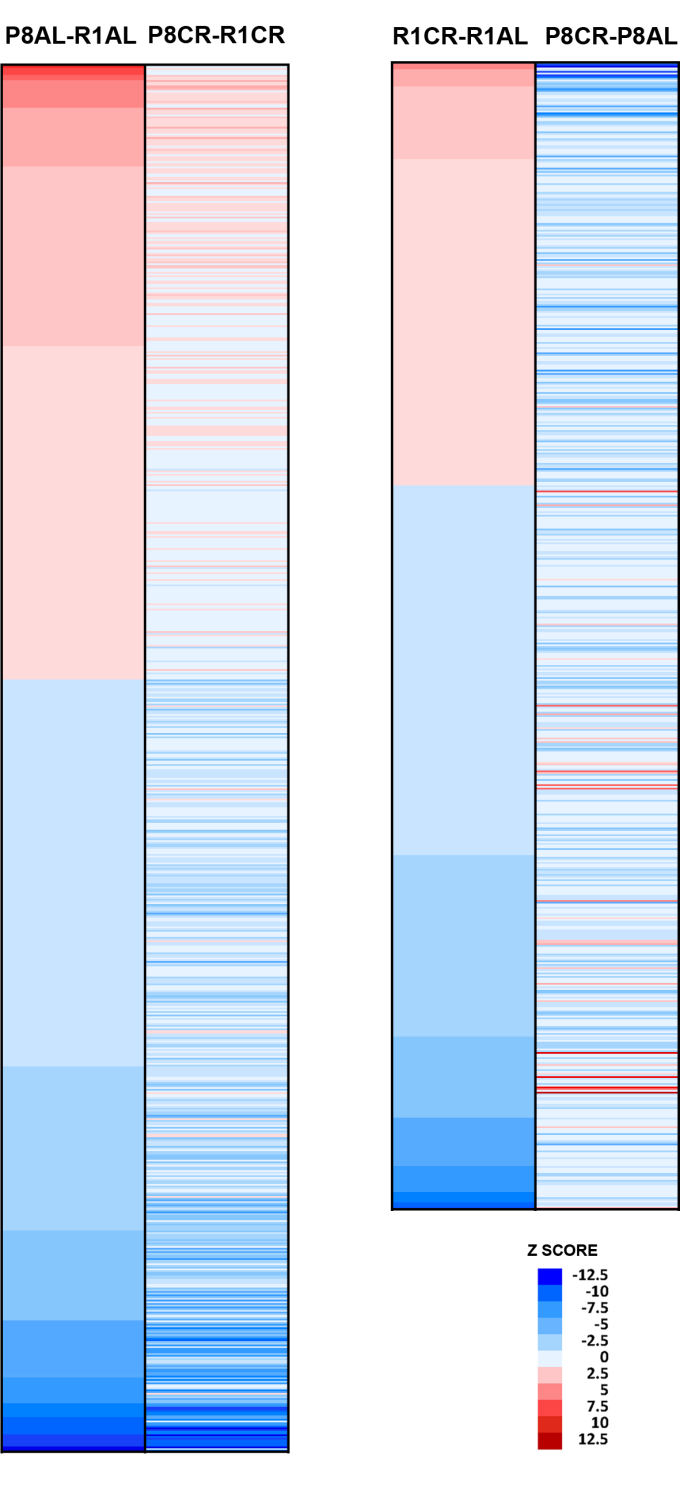
**

**Fig. S1** Caloric restriction brought the gene expression profile of the senescence astrocytes from SAMP8 mice closer to that of control astrocytes from SAMR1 mice. Parametric analysis of gene-set enrichment (PAGE) comparing every pathway that was significantly upregulated (red) or downregulated (blue) in SAMP8 astrocytes compared to SAMR1 with *ad libitum* treatment (P8AL-R1AL) showed smaller differences after caloric restriction (CR) (P8CR-R1CR). CR induced an extensive gene down-regulation in control astrocytes (R1CR-R1AL), but not in SAMP8 astrocytes (P8CR-P8AL), their changes being opposed in 53% of cases.
