# Supplementary material for: In vitro caloric restriction induces protective genes and functional rejuvenation in senescent SAMP8 astrocytes
Source: Aging Cell. 2015 Feb 25;14(3):334–44. doi: 10.1111/acel.12259 (PMC4406662; doi:10.1111/acel.12259)
Supplement: Supplementary file 2 [file acel0014-0334-sd2.docx]

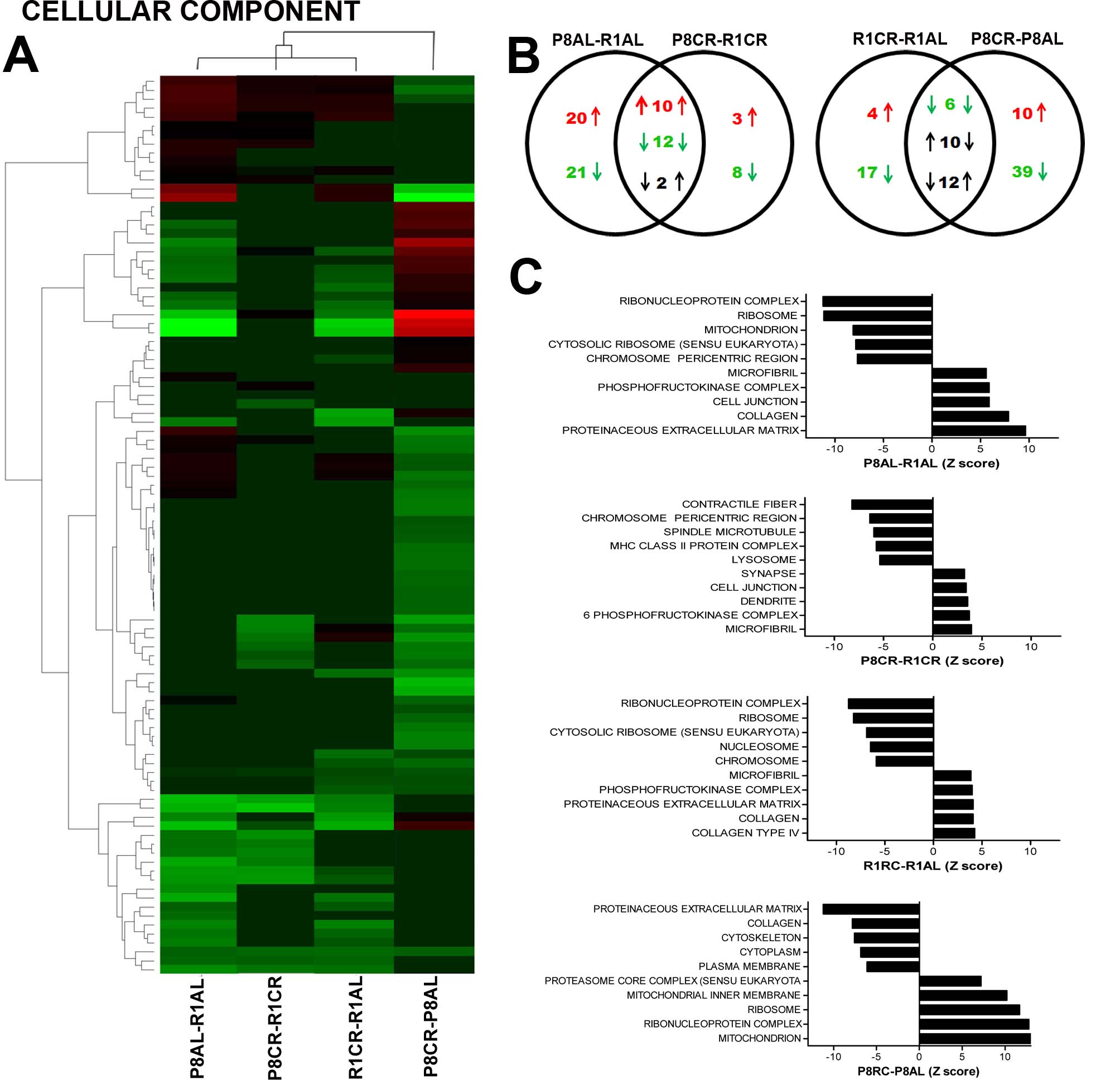


**Fig. S2** Gene pathways of cellular components were modulated differentially in SAMP8 versus SAMR1 astrocytes and these differences were attenuated by caloric restriction. (A) Hierarchical cluster of the 100 gene pathways of GeneOntology (GO) gene sets for cellular components most differentially modulated between the experimental groups. (B) Venn diagrams showing the number of pathways upregulated (upward arrow, red number), downregulated (downward arrow, green number) or with opposed changes (black number). (C) Histograms indicating the GO terms for the top five upregulated and downregulated cellular components between the experimental groups
