# Supplementary material for: In vitro caloric restriction induces protective genes and functional rejuvenation in senescent SAMP8 astrocytes
Source: Aging Cell. 2015 Feb 25;14(3):334–44. doi: 10.1111/acel.12259 (PMC4406662; doi:10.1111/acel.12259)
Supplement: Supplementary file 4 [file acel0014-0334-sd4.docx]

**
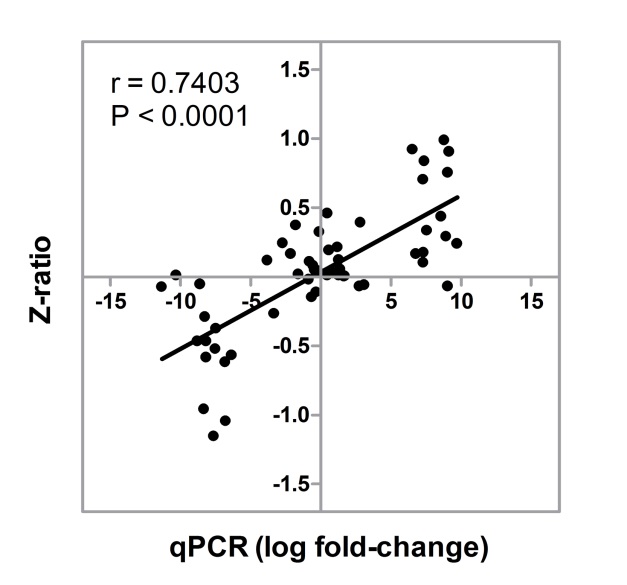
**

**Fig. S4 Correlation analysis between Z-ratio and real time qPCR fold-change of selected genes.** Pearson analysis indicated a high correlation between Z-ratio given by Illumina microarrays and fold changes obtained by RT-qPCR of selected genes differentially expressed in the senescent astrocytes SAMP8 compared to SAMR1 (See Table S6).
