# Supplementary material for: In vitro caloric restriction induces protective genes and functional rejuvenation in senescent SAMP8 astrocytes
Source: Aging Cell. 2015 Feb 25;14(3):334–44. doi: 10.1111/acel.12259 (PMC4406662; doi:10.1111/acel.12259)
Supplement: Supplementary file 10 [file acel0014-0334-sd10.docx]

**Table S6**. Real-time qPCR validation of microarray data: selected genes upregulated or downregulated in SAMP8 versus SAMR1.

| SAMP8:SAMR1 | Gene  symbol | Accession | | qPCR primer  sequences | P8AL-R1AL | | | | P8CR-R1CR | | | | R1CR-R1AL | | | | P8CR-P8AL | | | |  |
| --- | --- | --- | --- | --- | --- | --- | --- | --- | --- | --- | --- | --- | --- | --- | --- | --- | --- | --- | --- | --- | --- |
|  |  |  | | *Forward*  *Reverse* | Z ratio | | qPCR  fold- change | | Z ratio | | qPCR  fold- change | | Z ratio | | qPCR  fold- change | | Z ratio | | qPCR  fold- change | |  |
| Upregulated | Slc25a18 | NM_001081048.1 | ccctctggatgttctgaagact  ctgatctgttagtgtggcaagg | | 8.95 | | 1.95 ±  0.0058 | | 9.73 | | 1.73 ±  0.0052 | | 3.11 | | 0.87 ±  0.0009 | | -0.31 | | 0.77 ±  0.0023 | |  |
|  | Reck | NM_016678 | actcttctcctgggccatct  cagttgggtttctcattgga | | 7.57 | | 2.15 ±  0.0043 | | 8.60 | | 2.72 ±  0.0027 | | 1.71 | | 1.01 ±  0.0000 | | -0.79 | | 1.28 ±  0.0013 | |  |
|  | Glycam1 | NM_008134.2 | caaggagccttccatcttca  atcctgggcctcttgattct | | 7.4 | | 6.85 ±  0.1027 | | 9.17 | | 8.00 ±  0.0320 | | 2.78 | | 0.85 ±  0.0017 | | 1.68 | | 1.00 ±  0.0040 | |  |
|  | Gpr137b-ps | NR_003568.1 | atctgacctgtgctgtgctg  tgtcattgatggccactctc | | 7.32 | | 1.26 ±  0.0088 | | 9.07 | | 0.85 ±  0.0034 | | 1.2 | | 1.63 ±  0.0131 | | -0.19 | | 1.10 ±  0.0044 | |  |
|  | Ddit4l | NM_030143.3 | ctgggattatgttgtccctga  ttggtttgctttgatctgga | | 7.31 | | 5.03 ±  0.0402 | | 9.07 | | 5.65 ±  0.0339 | | 0.90 | | 1.07 ±  0.0011 | | -0.52 | | 1.20 ±  0.0072 | |  |
|  | Ndst1 | NM_008306.4 | gaggctgtgtcggcacct gcagagacaaaaacgctgaa | | 6.81 | | 1.46 ±  0.0205 | | 7.35 | | 1.49 ±  0.0089 | | 1.30 | | 1.02 ±  0.0031 | | -1.58 | | 1.04 ±  0.0063 | |  |
|  | Inmt | NM_009349.3 | ggagggcaaggtatacatagga  ggccagagtggaagctgtag | | 6.55 | | 8.33 ±  0.0001 | | 8.82 | | 9.71 ±  0.0068 | | 1.41 | | 1.13 ±  0.0000 | | 1.30 | | 1.32 ±  0.0009 | |  |
|  |  |  |  | |  | |  | |  | |  | |  | |  | |  | |  | |  |
| Downregulated | Gdpd5 | NM_201352.2 | gcaatactacgagccccaac  atcgtgcgagcgttggta | | -8.58 | | 0.88 ±  0.0008 | | -11.32 | | 0.84 ±  0.0004 | | -0.31 | | 1.07 ±  0.0005 | | 0.49 | | 1.02 ±  0.0244 | |  |
|  | Emb | NM_010330.3 | gggggattctactgtgctga  tgcggtttcattacccatgt | | -8.15 | | 0.34 ±  0.0000 | | -10.3 | | 1.02 ±  0.0004 | | -0.84 | | 0.95 ±  0.0038 | | 0.49 | | 2.87 ±  0.0057 | |  |
|  | Npy | NM_023456.2 | gtgtgtttgggcattctgg  tgtcgcagagcggagtagta | | -7.61 | | 0.07 ±  0.0000 | | -8.15 | | 0.26 ±  0.0002 | | -0.63 | | 0.71 ±  0.0014 | | 2.85 | | 2.46 ±  0.0015 | |  |
|  | Hspb1 | NM_013560.1 | gactggtaccctgcacacag  ctgaaccactgcgaccact | | -7.50 | | 0.30 ±  0.0012 | | -8.79 | | 0.34 ±  0.0030 | | -3.80 | | 1.31 ±  0.0052 | | -2.03 | | 1.46 ±  0.0131 | |  |
|  | Fcgr3 | NM_010188.4 | tgctcaaggaagacatggtg  ggatggaactccagttgtgg | | -7.45 | | 0.42 ±  0.0000 | | -8.24 | | 0.51 ±  0.0000 | | -2.70 | | 1.74 ±  0.0226 | | -0.09 | | 2.10 ±  0.0001 | |  |
|  | Gpnmb | NM_053110.3 | ccgatcacatgagagagcac  ccctcctccacactggatac | | -6.81 | | 0.24 ±  0.0005 | | -8.32 | | 0.11 ±  0.0002 | | -1.76 | | 2.35 ±  0.0986 | | -0.43 | | 1.12 ±  0.0022 | |  |
|  | Clec4d | NM_010819.3 | gacctgctgtcctgttagc  ctctcatgccaggtctggtt | | -6.76 | | 0.09 ±  0.0000 | | -6.33 | | 0.27 ±  0.0000 | | -3.31 | | 0.54 ±  0.0027 | | 0.6 | | 1.55  0.0002 | |  |
|  |  |  | |  | |  | |  | |  | |  | |  | |  | |  | |  | |

Note: P8AL-R1AL, difference between SAMP8 (P8) and SAMR1 (R1) astrocyte cultures in *ad libitum* (AL) condition; P8CR-R1CR, difference after caloric restriction (CR); R1CR-R1AL, changes after CR in SAMR1; P8CR-P8AL, changes after CR in SAMP8. Z ratio from the microarray analysis was calculated as described in Methods. Gene expression by real-time qPCR is expressed as mean ± SEM of fold changes of first indicated culture versus the second one, n=6-8; data were normalized to Gapdh values. See Table S7 for primer sequence of Gapdh.
