# Supplementary material for: In vitro caloric restriction induces protective genes and functional rejuvenation in senescent SAMP8 astrocytes
Source: Aging Cell. 2015 Feb 25;14(3):334–44. doi: 10.1111/acel.12259 (PMC4406662; doi:10.1111/acel.12259)
Supplement: Supplementary file 11 [file acel0014-0334-sd11.docx]

**Table S7**. Real-time qPCR validation of microarray data: representative genes of mitochondria and antioxidant defence upregulated in SAMP8 astrocytes after caloric restriction.

| Gene  symbol | Accession | | Name | | | qPCR primers | | | P8CR-P8AL | |
| --- | --- | --- | --- | --- | --- | --- | --- | --- | --- | --- |
|  | |  |  | | | *Forward*  *Reverse* | | | Z ratio | qPCR  fold-change |
| Ndufa1 | | NM_019443 | NADH dehydrogenase (ubiquinone) 1 alpha subcomplex, 1 | | | GTGTCCACTGCGTACATCCA  CAGGCCCTTGGACACATAGT | | | 3.75 | 1.249 ±  0.2446 |
| Uqcrh | | NM_025641 | Ubiquinol-cytochrome c reductase hinge protein | | | GGACTAGAGGACGAACGAAAGA  GGCCTTTACACACTTCTCCAG | | | 4.13 | 1.168 ±  0.1500 |
| Cox6b1 | | NM_025628 | Cytochrome c oxidase, subunit VIb polypeptide 1 | | | ACTACCTGGACTTCCACCG  ACCCATGACACGGGACAGA | | | 3.09 | 1.455 ±  0.1182 |
| Atp5E | | NM_025983 | ATP synthase, H+ transporting, mitochondrial F1 complex, epsilon subunit | | | GATGCCCTGAAGACCGAGTT  TTTTATGCTGCTGCCCGAAG | | | 3.70 | 1.576 ±  0.5384 |
| Mrpl14 | | NM_026732 | Mitochondrial ribosomal protein L14 | | | AAGCACTCATCGTGGGACAC  CACAGGGTTGCCATTGTCCT | | | 2.91 | 1.231 ±  0.1783 |
| Sod1 | | NM_011434 | Superoxide dismutase 1, soluble | | | CCACGTCCATCAGTATGGGG CGTCCTTTCCAGCAGTCACA | | | 3.06 | 1.125 ±  0.1620 |
| Gpx3 | | NM_008161 | Glutathione peroxidase 3 | | | GGTCAGGGGTGGTGTCTCTA  GCCTGAATGCACTAAGGGCT | | | 2.96 | 1.402 ±  0.1960 |
| Gpx8 | | NM_027127 | Glutathione peroxidase 8 (putative) | | | CTAGTGACTGCCGCTTCACA  GATAGGGCCCGAACTCCTTG | | | 3.28 | 1.117 ±  0.1310 |
| Atox1 | | NM_009720 | ATX1 (antioxidant protein 1) homolog 1 (yeast) | | | AACTGAAGAGGCAGGCTGTTG  TGTCTGGGCAGGAACTCCAT | | | 2.40 | 1.204 ±  0.1162 |
| Nudt1 | | NM_008637 | Nudix (nucleoside diphosphate linked moiety X)-type motif 1 | | | AGTCAGTGAGCTTCACACAGA  GGGAATGGATTGCTAGAGAGGAC | | | 2.38 | 1.213 ±  0.2744 |
| Gapdh | | NM_32599 | Glyceraldehyde-3-phosphate dehydrogenase | | | CACCCACTGCTTAGCCCC  TCTTCTGGGTGGCAGTGATG | | | -- | -- |
|  | |  | |  |  | |  |  |  |  |

Note: P8CR-P8AL, changes in SAMP8 (P8) astrocyte cultures after caloric restriction (CR) treatment as compared to *ad libitum* (AL) condition. Z ratio from the microarray analysis was calculated as described in Methods. Gene expression by real-time qPCR is expressed as mean ± SEM, n=3-4; data were normalized to Gapdh values. See also Fig. 2B.
